# Adoption of Mobile Apps for Depression and Anxiety: Cross-Sectional Survey Study on Patient Interest and Barriers to Engagement

**DOI:** 10.2196/11334

**Published:** 2019-01-25

**Authors:** Jessica Lipschitz, Christopher J Miller, Timothy P Hogan, Katherine E Burdick, Rachel Lippin-Foster, Steven R Simon, James Burgess

**Affiliations:** 1 Department of Psychiatry Brigham and Women's Hospital Boston, MA United States; 2 Department of Psychiatry Harvard Medical School Boston, MA United States; 3 Center for Healthcare Organization and Implementation Research VA Boston Healthcare System Boston, MA United States; 4 Center for Healthcare Organization and Implementation Research Edith Nourse Rogers Memorial Veterans Hospital Bedford, MA United States; 5 Division of Health Informatics and Implementation Science Department of Quantitative Health Sciences University of Massachusetts Medical School Worcester, MA United States; 6 Geriatrics and Extended Care Service Veterans Affairs Boston Healthcare System Boston, MA United States; 7 Harvard Medical School Boston, MA United States

**Keywords:** mHealth, depression, anxiety, mobile apps, patient preference

## Abstract

**Background:**

Emerging research suggests that mobile apps can be used to effectively treat common mental illnesses like depression and anxiety. Despite promising efficacy results and ease of access to these interventions, adoption of mobile health (mHealth; mobile device–delivered) interventions for mental illness has been limited. More insight into patients’ perspectives on mHealth interventions is required to create effective implementation strategies and to adapt existing interventions to facilitate higher rates of adoption.

**Objective:**

The aim of this study was to examine, from the patient perspective, current use and factors that may impact the use of mHealth interventions for mental illness.

**Methods:**

This was a cross-sectional survey study of veterans who had attended an appointment at a single Veterans Health Administration facility in early 2016 that was associated with one of the following mental health concerns: unipolar depression, any anxiety disorder, or posttraumatic stress disorder. We used the Veteran Affairs Corporate Data Warehouse to create subsets of eligible participants demographically stratified by gender (male or female) and minority status (white or nonwhite). From each subset, 100 participants were selected at random and mailed a paper survey with items addressing the demographics, overall health, mental health, technology ownership or use, interest in mobile app interventions for mental illness, reasons for use or nonuse, and interest in specific features of mobile apps for mental illness.

**Results:**

Of the 400 potential participants, 149 (37.3%, 149/400) completed and returned a survey. Most participants (79.9%, 119/149) reported that they owned a smart device and that they use apps in general (71.1%, 106/149). Most participants (73.1%, 87/149) reported interest in using an app for mental illness, but only 10.7% (16/149) had done so. Paired samples *t* tests indicated that ratings of interest in using an app recommended by a clinician were significantly greater than general interest ratings and even greater when the recommending clinician was a specialty mental health provider. The most frequent concerns related to using an app for mental illness were lacking proof of efficacy (71.8%, 107/149), concerns about data privacy (59.1%, 88/149), and not knowing where to find such an app (51.0%, 76/149). Participants expressed interest in a number of app features with particularly high-interest ratings for context-sensitive apps (85.2%, 127/149), and apps focused on the following areas: increasing exercise (75.8%, 113/149), improving sleep (73.2%, 109/149), changing negative thinking (70.5%, 105/149), and increasing involvement in activities (67.1%, 100/149).

**Conclusions:**

Most respondents had access to devices to use mobile apps for mental illness, already used apps for other purposes, and were interested in mobile apps for mental illness. Key factors that may improve adoption include provider endorsement, greater publicity of efficacious apps, and clear messaging about efficacy and privacy of information. Finally, multifaceted apps that address a range of concerns, from sleep to negative thought patterns, may be best received.

## Introduction

The majority of the US population owns smartphones (77% in 2016) [1], and the number of mobile apps for health has grown exponentially over the past decade. A study by the IMS Institute for Healthcare Informatics [2] found that the number of health and wellness apps available to consumers has more than doubled between 2013 and 2015 (from 43,000 to over 90,000). Although the content and quality of these apps vary widely, the potential public health impact of such tools is enormous. Research suggests that mobile health (mHealth) interventions can have a positive influence on a wide range of health conditions [3,4] and, while not a substitute for in-person treatment, these tools offer a treatment option that does not have as many access barriers as in-office treatment (eg, no transportation is required) and may allow for reduced cost of care (since marginal cost is negligible).

In mental illness—where stigma and self-reliance beliefs are additional barriers to treatment seeking and engagement [5]—mobile health (mHealth) offers even greater potential. Common mental health disorders such as depression and anxiety impact nearly a third of the US population, and most of those who need treatment do not receive it [6,7]. The sheer number of people affected makes providing adequate treatment in traditional clinical settings prohibitive in terms of availability of trained providers. Studies indicate that mHealth interventions can improve functioning and symptoms in those with depression and/or anxiety [8-11] and also that technology offers some advantages over in-person treatments. Specifically, mHealth interventions offer 24/7 support because mobile devices are often kept with users throughout the day. In addition, patients may be more likely to report severe symptoms on technology platforms than in person [12], and patients value the autonomy and empowerment that can be offered by such platforms [13].

Unfortunately, adoption of mHealth interventions for common mental illnesses such as depression and anxiety remains low. To date, mHealth is neither a routine part of mental health care offerings in the United States nor has any mHealth platform for mental illness been widely adopted by consumers in the United States. These patterns are particularly noteworthy in systems such as the Veterans Health Administration (VHA), which has invested substantially in building and evaluating several free behavioral health apps specifically designed for mental health concerns of veterans. Several theoretical models explaining technology adoption and continued use have been put forth in the literature [14-16]. Existing models have some conflicting and some overlapping components and have been found to explain as little as 17% and as much as 53% of the variance in adoption [15]. Newer unified models may explain more of the variance in adoption and use, but much of this literature has traditionally focused largely on adoption of technology in the workplace, a considerably different context than the treatment of mental illness. This multifaceted theoretical canvas underscores the complexity of understanding adoption and the potential importance of studying specific types of technology within the intended use population. At present, it is unclear what are the best approaches for encouraging patient adoption of mHealth interventions.

Research on patient adoption of technology in treatment of mental illness suggests that interest outpaces adoption. Specifically, studies of patients with depression, anxiety, and posttraumatic stress disorder (PTSD) suggest that interest varies widely based on the type of technology in question, but most patients are interested in using some kinds of technology in treatment [17,18]. With regard to mHealth specifically, Erbes et al [19] found that over half of a sample of patients with PTSD expressed interest in mHealth programs for PTSD, but less than 10% were currently using these platforms to help manage their symptoms.

Given high interest and low adoption, there is a need to build a stronger understanding of the factors that may affect adoption at the system level. Research on other patient-facing technologies suggests that how such technologies are integrated into the health care system may impact patient adoption. For example, findings from studies focused on adoption of one Web portal indicate that provider endorsement can improve rates of adoption [20]. It remains to be determined whether this is the case for mHealth interventions.

There is also a need to build a stronger understanding of factors that may affect adoption at the patient level. A large national survey of health app use in the general population indicated that lack of interest, cost, and concern about data privacy were key barriers to adoption [21]. These findings have been reinforced in other studies focused on mental health apps. Specifically, a study focused on mHealth interventions for depression found that cost, concerns about privacy, concerns over intervention efficacy, and misfit of intervention features to needs (ie, personalization) were key barriers to adoption of depression apps [22]. Another study focused on health and mental health apps found that efficacy and privacy are key barriers to adoption as well as not knowing where to find an app or knowing which app to download [23]. However, these studies were conducted using only partially clinical samples, that is, presence of clinically significant symptoms (on self-report or via medical record diagnosis) was either not an eligibility criterion or not assessed.

Stronger understanding of patient perspectives on mHealth interventions in relevant clinical samples is required to support the development of targeted implementation strategies and platform modifications that will ultimately promote adoption. The aim of this study was to characterize mHealth interest, concerns, and preferences in a sample of patients with an active diagnosis of depression, anxiety, and/or PTSD. Specifically, we sought to (1) identify patients’ degree of interest in mHealth interventions for mental health, (2) identify whether provider endorsement would impact degree of interest, (3) determine reasons for nonuse of mHealth interventions for mental health, and (4) identify what mHealth content or features are of most interest to patients.

## Methods

### Recruitment

We used the Veterans Affairs (VA) Corporate Data Warehouse (CDW) to identify individuals meeting eligibility criteria and to extract contact and diagnostic information for those individuals. Eligibility criteria were as follows: (1) US military veteran enrolled in care at the VA Boston Healthcare System; (2) receiving VA primary care, as indicated by having at least one encounter in the local primary care clinic between January 1, 2016, and July 1, 2016; (3) aged 18 years or older; and (4) attended a VA medical appointment between January 1, 2016, and July 1, 2016, in which an anxiety disorder (including obsessive-compulsive disorder), unipolar depressive disorder, or PTSD was documented as a condition treated in the appointment. Codes based on the 10th revision of the International Statistical Classification of Diseases and Related Health Problems (ICD-10) were used to determine visits associated with unipolar depression (F32-F34) and anxiety and PTSD (F40-F43). The decision to include patients with any or all of these diagnoses in the sample was based on high comorbidity rates between these diagnoses and the similarity of pharmacological and psychotherapeutic treatments for these disorders [24-29].

A total of 2840 veterans in the CDW met the above criteria. Within this sample, we divided records into 4 strata (white men, nonwhite men, white women, and nonwhite women) and randomly sampled 100 records from each stratum to achieve a gender- and minority-balanced set of potential participants. These randomly selected 400 individuals were actively recruited for participation via mailed surveys and accompanying study information. Although electronic medical record diagnostic codes were used to define our CDW search parameters and establish a set of eligible participants, these codes were not extracted for use in our dataset. This decision was made to protect patients’ privacy, especially those patients who chose not to participate. The only information extracted from patient’s charts was name and mailing address.

We used a modified Dillman method for recruitment [30]. The 400 veterans identified as potential participants were sent a series of 3 mailings, each including a letter inviting the veteran to participate, a study fact sheet, the survey, a postage-marked opt-out postcard, and a postage-marked return envelope. In addition, the first mailing contained a $10 Patron coupon for use at the local VA facility cafeteria and general store. The study invitation letter informed veterans that they may keep this coupon regardless of their decision to participate in this research. Participants who returned either the survey or opt-out postcard were not included in successive mailings.

All recruitment and study procedures were approved by the VA Boston Healthcare System’s institutional review board.

### Survey

Survey items were a combination of validated measures and newly developed questions based on the literature on technology use and adoption [31-33]. As there was no precedent for items evaluating concerns related to mental health app use and/or interest after clinician endorsement, these items were developed based on existing literature and field tested among a diverse team of colleagues with expertise in survey development. Items on mental health app features of interest to participants were selected based on a review of the literature on common elements of depression and anxiety apps [34,35].

The final survey consisted of 38 questions focused on 6 domains: (1) sociodemographic characteristics; (2) physical and mental health symptoms assessed using the SF-1 (first item of the 36-item Short Form Health Survey) for overall health [31,36], the Patient Health Questionnaire-8 (PHQ-8) for depression symptom severity [32,37], and Generalized Anxiety Disorder-7 (GAD-7) for anxiety symptom severity [33,38,39]; (3) technology ownership and use; (4) interest in apps for mental illness; (5) reasons for not using apps for mental illness; and (6) interest in specific mental illness app features (see Multimedia Appendix 1 for a list of items in each domain).

### Data Analysis

We aggregated descriptive data on the following: demographic and health characteristics, devices owned, current technology use, and ratings on interest in mHealth interventions.

We used paired sample *t* tests to evaluate the degree to which provider endorsement impacted participants’ level of interest in use of mHealth interventions for mental illness. Specifically, *t* tests compared participants’ general interest ratings with those provided when asked how interested they would be in using a mobile app for mental illness if their primary care provider (PCP) recommended it. A similar comparison was conducted between general interest ratings and those provided when asked how interested they would be in using a mobile app for mental illness if their mental health provider recommended the app. Finally, we used *t* tests to compare interest ratings associated with PCP recommendation with those associated with mental health provider recommendation.

We also compiled aggregate descriptive data on the following: reasons endorsed for using or not using mobile apps for mental health and interest in specific app features and content.

## Results

### Participants

A total of 149 surveys were returned (response rate of 37.3%, 149/400). The resulting sample was fairly balanced on demographic characteristics (see Table 1). For clarity and because no item or scale had missing data for more than 8.1% (12/149) of respondents, all results are reported as percentages of the full sample.

The mean PHQ-8 score was 11.25 (SD 6.62), and the majority of the sample (65.8%, 98/149) reported symptoms that met the PHQ-8 cutoff score of 8, indicating clinically significant depressive symptoms [40]. The mean GAD-7 score was 9.65 (SD 6.02), and more than half of the sample (56.4%, 84/149) reported symptoms that met the GAD-7 cutoff score of 8 for clinically significant anxiety symptoms [33,39]. Self-reported mental health conditions were collected and are detailed in Table 1.

### Technology Ownership and Use

The majority of the participants reported owning a smartphone (75.8%, 113/149) and a smaller portion reported owning a tablet (45.6%, 68/149). Together, a total of 119 participants (79.9%, 119/149) reported owning a smart device that could be used to run a mental health app. Table 2 displays participant answers with regard to current app and smart device technology use.

### Interest in Apps for Mental Illness

When asked how interested they would be in using an app for mental illness, 73.1% (87/149) reported some level of interest. Specifically, 12.8% (19/149) indicated that they would be completely interested, 22.1% (33/149) indicated that they would be very interested, 22.8% (34/149) indicated that they would be moderately interested, and 15.4% (23/149) indicated that they would be a little interested. When the sample was limited to only those who owned a smart device, the percentage of individuals with some level of interest in using an app for mental illness was slightly higher (77.3%, 92/149).

In addition, when asked about interest in apps that could deliver context-sensitive feedback (ie, utilizing passive sensors to respond to physical or behavioral changes), the majority of the sample (84.0%, 125/149) reported some interest. Specifically, 28.9% (43/149) reported that they would be completely interested, 26.2% (39/149) reported that they would be very interested, 16.1% (24/149) reported that they would be moderately interested, and 12.8% (19/149) reported that they would be a little interested. When the sample was limited to only those who owned a smart device, the percentage of individuals interested in an app that delivered context-sensitive feedback was only slightly higher (86.6%, 103/149).

### Relationship Between Interest in Apps for Mental Illness and Provider Endorsement

Paired sample *t* tests were used to determine whether provider endorsement would impact interest levels*.* Starting with an alpha=.05 as the critical P value, the Bonferroni corrected P value for 3 t tests was .017. Participants rated global interest independent of provider endorsement (mean 2.81 [SD 1.38]) significantly lower than interest in the context of PCP endorsement (mean 3.13 [SD 1.38], *t*_147_=−5.65, *P*<.001, *d*=0.23). Similarly, participants rated global interest independent of provider endorsement (mean 2.81 [SD 1.38]) significantly lower than interest in the context of mental health provider endorsement (mean 3.30 [SD 1.36], *t*_145_=−4.05, *P*<.001, *d*=0.36). Finally, participants rated interest in the context of PCP endorsement (mean 3.13 [SD 1.38]) significantly lower than interest in the context of mental health provider endorsement (mean 3.30 [SD 1.36], *t*_145_=−3.37, *P*<.001, *d*=0.12). When the sample was limited to only those who owned smart devices (n=119), these comparisons remained significant at the *P*<.001 level in the same directions.

### Reasons for Not Using Apps for Mental Illness

Table 3 displays the frequency with which participants endorsed specific reasons for not using mental health apps. The most commonly endorsed reasons were not having proof that the app would work, concerns about privacy, and not knowing where to find such an app. These were the most commonly endorsed reasons both when the full sample was considered and when the sample was limited to only those participants who owned smart devices.

### Interest in Specific Mental Illness App Features

Table 4 displays the frequency with which participants endorsed interest in features of mental health apps. The features with the highest interest ratings related to increasing exercise, getting better sleep, cognitive restructuring (changing negative or self-critical thinking), and behavioral activation (getting involved in more activities). These features were the most frequently endorsed both when the full sample was considered and when the sample was limited to only those participants who owned smart devices.

**Table 1:** Demographic characteristics of the sample (N=149).

Characteristics		Statistics
Age (years), mean (SD)		57.5 (13.9)
**Gender, n (%)**		
	Male	77 (51.7)
	Female	67 (45.0)
	Not reported	5 (3.4)
**Race or ethnicity, n (%)**		
	Caucasian or white	67 (45.0)
	African American or black	44 (29.5)
	Other	11 (7.4)
	Hispanic or Latino	9 (6.0)
	Not reported	7 (4.7)
	Asian	6 (4.0)
	American Indian, Alaskan Native	4 (2.7)
	Pacific Islander	1 (0.7)
**Education, n (%)**		
	Middle school (7th-8th)	1 (0.7)
	High school (9th-12th)	24 (16.1)
	Some college or vocational school	41 (27.5)
	Associates degree (2-year college)	16 (10.7)
	Bachelor’s degree (4-year college or university)	36 (24.2)
	Graduate degree	27 (18.1)
	Not reported	4 (2.7)
English as first language, n (%)		134 (89.9)
**Marital status, n (%)**		
	Divorced or separated	49 (32.9)
	Married	46 (30.9)
	Single, never married	39 (26.2)
	Widowed	11 (7.4)
	Not reported	4 (2.7)
**Annual household income, n (%)**		
	Less than US $20,000	36 (24.2)
	US $20,000 to US $34,999	21 (14.1)
	US $35,000 to US $49,999	35 (23.5)
	US $50,000 to US $74,999	20 (13.4)
	US $75,000 to US $99,999	15 (10.1)
	US $100,000 to US $149,999	8 (5.4)
	US $150,000 or more	2 (1.3)
	Not reported	12 (8.1)
**Self-reported health rating, n (%)**		
	Excellent	3 (2.0)
	Very good	21 (14.1)
	Good	56 (37.6)
	Fair	51 (34.2)
	Poor	11 (7.4)
	Not reported	6 (4.0)
**Self-reported behavioral health conditions, n (%)**		
	Depression	107 (71.8)
	Stress	97 (65.1)
	Anxiety	96 (64.4)
	Difficulty sleeping	93 (62.4)
	Posttraumatic stress disorder	91 (61.1)
	Chronic pain	88 (59.1)
	Overweight	76 (51.0)
	Smoking	32 (21.5)
	Diabetes	26 (17.4)
	Substance use disorder (not alcohol)	15 (10.1)
	Alcohol use disorder	14 (9.4)

**Table 2:** Technology use characteristics of sample (N=149).

Type of technology use		Frequency endorsed, n (%)
**Smartphone or tablet functions**		
	Texting	118 (79.2)
	Taking pictures or camera	116 (77.9)
	Apps	106 (71.1)
	Searching the internet	104 (69.8)
	Checking the weather forecast	103 (69.1)
	Email	101 (67.8)
	Driving or walking directions	95 (63.8)
	Social media	83 (55.7)
**Use of apps for other health-related goals**		
	Daily steps	42 (28.2)
	Tracking calories	34 (22.8)
	Mindfulness exercises	31 (20.8)
	Weight management	30 (20.1)
	Sleep	28 (18.8)
	Mental illness	16 (10.7)

**Table 3:** Factors impacting use of mental health apps.

Reason	Smart device owners (n=119), n (%)	Full sample (N=149), n (%)
I might use an app for these problems if I saw proof that it worked.	92 (77.3)	107 (71.8)
I am concerned about protecting my privacy with having my information in an app like this.	73 (61.3)	88 (59.1)
I don’t know how to find an app that would help.	61 (51.3)	76 (51.0)
I don’t think an app can help me to get better.	44 (37.0)	55 (36.9)
I am already in treatment for stress, depression, anxiety or PTSD^a^ and don’t see the need for an app.	43 (36.1)	52 (34.9)
It would be embarrassing to have an app like this on my phone.	31 (26.1)	39 (26.2)
I don’t use apps at all.	13 (10.9)	29 (19.5)
I tried an app like this before and did not like it because it was not personalized enough.	13 (10.9)	14 (9.4)
I don’t think I have a problem with stress, depression, anxiety or PTSD.	12 (10.1)	21 (14.1)
I tried an app like this before and it did not help.	11 (9.2)	11 (7.4)
I tried an app like this before and did not like it because it was difficult to use.	10 (8.4)	12 (8.1)

^a^PTSD: posttraumatic stress disorder.

**Table 4:** Interest in specific features of mental health apps.

Item wording (intervention label)	Smart device owners (n=119), n (%)	Full sample (N=149), n (%)
Increase your physical activity or exercise (physical activity)	95 (79.8)	113 (75.8)
Help you learn to get better sleep (Cognitive Behavioral Therapy for Insomnia)	87 (73.1)	109 (73.2)
Learn how to change negative/self-critical thinking (cognitive restructuring)	86 (72.3)	105 (70.5)
Get involved in more activities (behavioral activation)	86 (72.3)	100 (67.1)
Track mood/stress/anxiety/PTSD^a^ symptoms (progress monitoring)	80 (67.2)	95 (63.8)
Speak with a health coach when your symptoms are bad. (professional support)	79 (66.4)	98 (65.8)
Learn more about your mental health condition. (psychoeducation)	77 (64.7)	92 (61.7)
Help improve your social skills (social skills training)	75 (63.0)	92 (61.7)
Remind you to take your medications. (medication adherence)	73 (61.3)	91 (61.1)
Connect with a community of people with similar mental health problems (social support)	61 (51.3)	72 (48.3)

^a^PTSD: posttraumatic stress disorder.

## Discussion

### Principal Findings

Results from this study indicate that access and interest in mobile apps for mental illness outpace actual use. Specifically, we found that access to devices and use of apps, in general, was high: nearly 80% of our sample reported owning smart devices, and of those with smart devices, nearly 90% reported that they use apps. Interest in using mobile apps for mental illness was also high: over 70% of the sample indicated that they have some level of interest. Despite owning the requisite devices, having active and relevant diagnoses (as indicated by PHQ-8 and GAD-7 scores), and expressing interest, use of mobile apps for mental illness was low: only 1 in 10 participants used apps for mental illness. These findings could be interpreted as indicating that most participants wanted to use mHealth interventions for mental illness and had the device and technology knowledge to do so.

Findings also provide some guidance into factors that may impact adoption. First, the highest-rated reasons for not using apps for mental health were related to not having proof of efficacy, concerns about whether these apps could keep mental health information adequately private, and not knowing where to find such an app. These findings suggest that public dissemination of information on efficacy of apps for mental illness (eg, in doctors’ offices or on public transportation) could improve adoption. Moreover, informing users how information within the app is protected (eg, in the introductory screens of the app) may increase adoption. Concerns related to efficacy and privacy are supported by earlier studies [21,22,41], but until recently [23], lack of information on where to find evidence-based apps has not been clearly articulated as a barrier to adoption. With regard to barriers to adoption, it is important to specifically note that this study did not evaluate cost as a barrier to adoption for 2 reasons. First, within VA, cost concerns of medical care are different than outside VA. Second, VA has developed a number of mobile apps for mental illness that are freely available to the public and relevant for the veterans recruited in this study.

Provider endorsement also appears to be a promising avenue for increasing adoption of mHealth for mental illness. Participants provided significantly higher interest ratings in the context of provider endorsement than when asked more generally about interest in using such apps. These findings are consistent with existing literature on the impact of provider endorsement in patient adoption of other patient-facing technologies (eg, patient portals that offer messaging and other features) [20]. These findings go beyond the existing literature, however, by showing that the type of provider endorsing the intervention may matter because interest ratings were greater in the context of mental health provider endorsement than PCP endorsement. Provider recommendation is not currently the norm; recent research suggests that individuals are more likely to hear about mental health apps through social media, Web searches, or friends than through medical providers [23]. Findings from our study underscore that providers could potentially play a key role in increasing adoption. Findings also raise questions about who among providers should be endorsing mHealth interventions to maximize the chances of adoption.

Although this study did not seek to directly test existing models of technology adoption, some interesting parallels between these findings and existing models were observed. Specifically, the Unified Theory of Acceptance and Use of Technology [15] indicates that 2 key determinants of technology adoption and use are performance expectancy (a user’s beliefs on whether the technology will be helpful) and social influence (how strongly an individual believes that important others think he or she should use the technology). Findings that both proof of efficacy and provider endorsement would encourage use are consistent with these 2 theoretical constructs. Considering the results from this research in relation to such constructs is particularly important to understanding how evolving theories of technology adoption can best be applied in different contexts, including patient adoption of technology and its integration into mental health treatment.

Findings also provide insight into what features and content of apps patients with depression, anxiety, and/or PTSD may find most useful. Over 70% of participants with smart devices reported interest in using apps that facilitate core functions of cognitive behavioral therapy such as cognitive restructuring and behavioral activation. Over 73% of participants with smart devices reported interest in features that would promote wellness in areas of behavioral health such as sleep difficulties and inactivity. These findings suggest that this population may be best served by individual apps or suites of apps that target depression and anxiety from multiple angles [10].

In addition, interest in context-sensing mobile app interventions was high; 85% of participants indicated some level of interest in this type of intervention. This finding contrasts with other research where participants endorsed skepticism and concern over context sensing [41]. Interest in context-sensing mobile app interventions may indicate an interest in personalization. Along these lines, Table 3 shows that the majority of those who reported having used an app for mental health also endorsed that they did not like it because it was not sufficiently personalized. This finding should be interpreted with caution because we do not know which apps these participants used, and it is difficult to draw conclusions based on such a small subsample (only 10.7% of the full sample had used apps for mental illness). However, other research corroborates that patient reports of insufficient personalization is a perceived barrier to using mobile treatment apps for depression [22].

It was also worth noting that although participants endorsed interest in apps that offered the option of speaking to a health coach, 5 other features were endorsed more frequently than this feature. There has been a lot of emphasis on the integration of health coaching into app platforms both as a way to enhance engagement and as a way to produce higher levels of change [42,43]. On the other side of this debate, some research indicates that integrating health coaching does not necessarily ensure engagement in technology-based interventions for depression as users can simply ignore calls from coaches [44]. Findings from this study contribute to this debate and indicate that health coaching capabilities may not be essential for user interest and/or engagement.

### Strengths and Limitations

Key strengths of this study include engagement of a racially diverse, clinical sample and proactive recruitment methods. By mailing paper surveys to patients identified as eligible, we expect to have captured data from individuals who may not have responded to more passive recruitment approaches (eg, flyers in waiting rooms). However, our proactively mailed survey methodology also introduces some bias as it is also possible that those who were less interested in use of technology were less likely to respond to the survey. Nevertheless, it is our expectation that the clinical nature of our sample was appropriate for our research questions and that our recruitment method introduced less bias than studies recruiting online or via social media, which essentially make technology proficiency a condition for entry into the study.

The sample in this study consisted entirely of veterans receiving services at a single VA hospital in a metropolitan area in the northeastern United States. Generalizability of findings to nonveteran samples and samples collected in other geographical areas should be tested in future studies. In addition, given the scope and funding level of this study, the presence of diagnoses required for eligibility was based on patients’ medical records and not verified by study staff independently through a structured clinical interview.

Finally, this study evaluated stated preferences and interests. A close-ended question format was used for this survey; however, the downside of survey items formatted in this manner is that they can produce less nuanced data when answer options do not fully capture patients’ thoughts. Additional research that includes more nuanced data collection such as a mixed-methods study with qualitative interviews will be an important next step. Moreover, moving forward, it will be necessary to evaluate whether these self-reported findings hold up behaviorally. That is, future research will need to assess whether implementation strategies and platforms consistent with observed preferences and interests are associated with positive impact on adoption and engagement.

### Conclusions and Future Directions

Mobile apps are a new and promising adjunctive, and possibly even stand-alone, treatment option for patients with depression and anxiety disorders. They are technologies that can reach patients beyond the confines of traditional brick-and-mortar clinic visits and engage them directly, in the context of their daily lives. For these reasons, mobile apps are also a unique treatment option to implement, one that requires a thorough understanding of patient perspectives and preferences if effective implementation strategies are to be designed. As reinforced in this study, smart devices are ubiquitous and patients are interested in using this technology. Findings from this study offer several key takeaway points. First, in this sample of individuals with clinically significant mood and/or anxiety symptoms, most were interested in using mobile apps as part of treatment, but few were doing so. Second, participant interest ratings suggest that provider endorsement may positively influence adoption of these technologies. Third, integration of wearables and passive data to direct interventional content, interventions to improve self-care around sleep and inactivity, and common cognitive-behavioral therapy interventions such as cognitive restructuring and behavioral activation were all perceived as valuable by patients. Finally, messaging around these technologies should increase awareness of mobile apps available for this population, relay what is known around efficacy, and address privacy concerns. One way to disseminate these messages could be through patients’ providers, but this would require that providers have easy access to up-to-date information on which apps are efficacious and safe.

Evaluating the generalizability of these findings in a nonveteran sample and determining whether preferences observed here translate to actual behaviors will be critical moving forward. It will also be important to evaluate whether patient interest and concerns are different across various demographic subgroups (eg, gender, race, age, and education) to determine how best to create systems that meet the needs of all segments of the population. Adjusting messaging and implementation strategies in ways that reflect these findings and evaluating patient adoption and engagement are essential next steps. In addition, evaluating whether preferences endorsed translate to preferential use of specific app features in real-world settings could direct attention of app developers toward the features that patients most value.

## Appendix

Multimedia Appendix 1 Survey questions.

## Abbreviations

CDW: Corporate Data Warehouse
GAD-7: Generalized Anxiety Disorder-7
mHealth: mobile health
PTSD: posttraumatic stress disorder
PCP: primary care provider
PHQ-8: Patient Health Questionnaire-8
VA: Veteran Affairs
VHA: Veterans Health Administration
