# Supplementary material for: Adoption of Mobile Apps for Depression and Anxiety: Cross-Sectional Survey Study on Patient Interest and Barriers to Engagement
Source: JMIR Ment Health. 2019 Jan 25;6(1):e11334. doi: 10.2196/11334 (PMC6367667; doi:10.2196/11334)
Supplement: Multimedia Appendix 1 [file mental_v6i1e11334_app1.pdf]

APPENDIX: Survey Questions

Domain 1: Sociodemographic Characteristics

What is your age? ________ years

What is your gender?
☐ Male
☐ Female
☐ Other (please specify)

Which race/ethnicities describe you? *(Check all that apply.)*
☐ African American/Black
☐ Asian
☐ Caucasian/White
☐ Hispanic/Latino
☐ Pacific Islander
☐ American Indian, Alaskan Native
☐ Other_____________________

What is your highest level of education?
☐ Elementary school (1st-6th grade)
☐ Middle school (7th-8th grade)
☐ High school (9th-12th grade)
☐ Some college/vocational school
☐ Associate’s degree (2-year college)
☐ Bachelor’s degree (4-year college or university)
☐ Graduate degree
☐ Other ______________________

Is English your first language?
☐ Yes
☐ No

What is your current marital status?
☐ Single, never married
☐ Married
☐ Divorced/separated
☐ Widowed

What is your annual household income?
☐ Less than $20,000
☐ $20,000 to $34,999
☐ $35,000 to $49,999
☐ $50,000 to $74,999
☐ $75,000 to $99,999

☐ $100,000 to $149,999
☐ $150,000 to $199,999
☐ $200,000 or more

Domain 2: Physical and Mental Health Symptoms

Do you currently have any of the following health conditions?

Yes	No	
☐	☐	Depression
☐	☐	Anxiety
☐	☐	PTSD
☐	☐	Alcohol Problems
☐	☐	Drug Problems
☐	☐	Difficulty Sleeping
☐	☐	Overweight
☐	☐	Stress
☐	☐	Diabetes
☐	☐	Smoking
☐	☐	Chronic Pain

Would you say your health in general is:
☐ Excellent
☐ Very Good
☐ Good
☐ Fair
☐ Poor

Over the last 2 weeks, how often have you been bothered by any of the following problems?

	**Not at all**	**Several Days**	**More Than Half the Days**	**Nearly Every Day**
1. Little interest or pleasure in doing things	0	1	2	3
2. Feeling down, depressed or hopeless	0	1	2	3
3. Trouble falling or staying asleep, or sleeping too much	0	1	2	3
4. Feeling tired or having little energy	0	1	2	3
5. Poor appetite or overeating	0	1	2	3
6. Feeling bad about yourself, or that you are a failure, or have let yourself or your family down	0	1	2	3
	0	1	2	3

7. Trouble concentrating on things, such as reading the newspaper or watching television.				
8. Moving or speaking so slowly that other people could have noticed. Or the opposite – being so fidgety or restless that you have been moving around a lot more than usual.	0	1	2	3
9. Feeling nervous, anxious, or on edge	0	1	2	3
10. Not being able to stop or control worrying	0	1	2	3
11. Worrying too much about different things	0	1	2	3
12. Trouble relaxing	0	1	2	3
13. Being so restless that it's hard to sit still	0	1	2	3
14. Becoming easily annoyed or irritable	0	1	2	3
15. Feeling afraid as if something awful might happen	0	1	2	3

16. If you checked off any problems above, how difficult have these made it for you to do your work, take care of things at home, or get along with other people?

    ☐ Not difficult at all
    ☐ Somewhat difficult
    ☐ Very difficult
    ☐ Extremely difficult

Domain 3: Current Technology Ownership and Use

For the questions below please use the following definitions:

Smartphone refers to a cell phone, such as an iPhone or Android, that allows you to connect to the internet, send and receive emails, and/or use applications or "apps")

Tablet refers to a mobile computer with a touch screen that is smaller than a laptop but larger than a smartphone. Examples are the iPad, Kindle Fire or Surface.

Apps refer to programs or applications designed for use on a mobile device, like a smartphone or tablet. Apps typically appear as a small picture on the main screen. Examples of apps are Facebook, Instagram, Google Maps, Twitter and Gmail.

Do you have a **smartphone?**
☐ Yes
☐ No

Do you have a **tablet?**
☐ Yes
☐ No

Do you ever use **apps** on your smartphone or tablet?
☐ Yes
☐ No *(Check 'no' if you do not own a smartphone or tablet.)*

Do you use your phone or tablet for any of the following?

Yes	No	
☐	☐	Texting
☐	☐	Email
☐	☐	Driving/walking directions
☐	☐	Searching the internet
☐	☐	Camera functions (to take pictures or videos)
☐	☐	Social media (like Facebook, Instagram or Twitter)
☐	☐	Checking the weather forecast

Have you ever used an app to help you track or complete the following activities?

Yes	No	
☐	☐	Daily Steps
☐	☐	Diet (to track your calories or what you eat)
☐	☐	Weight
☐	☐	Mindfulness exercises
☐	☐	Sleep

Have you ever used an app to help you manage stress, depression, anxiety or PTSD**?**

☐ Yes
☐ No

Domain 4: Interest in Mental Health App Use

Please <u>read</u> the questions below and <u>circle</u> the number that best represents your opinion:

	**Not at all**	**A little**	**Medium**	**Very**	**Completely**
How interested are you in using an app to help you manage stress, depression, anxiety or PTSD?	1	2	3	4	5
How willing would you be to use an app to help you manage stress, depression, anxiety or PTSD ***if your <u>primary care provider</u> recommended the app***?	1	2	3	4	5
How willing would you be to use an app to help you manage stress, depression, anxiety or PTSD ***if your <u>mental health provider</u> (therapist or psychiatrist) recommended the app***?	1	2	3	4	5
How interested would you be in an app that could ***respond to physical (e.g., heart rate) or behavioral changes to know when you might***	1	2	3	4	5

be experiencing symptoms of stress, anxiety, depression or PTSD and suggest ways to manage symptoms?					

Domain 5: Reasons for Choosing to Use/Not Use Mental Health Apps

Below are some reasons people may **choose not to use** mobile apps for stress, depression, anxiety or PTSD. Which reasons are true for you?

Yes	No	
☐	☐	**I** I don't think an app can help me to get better.
☐	☐	I don't know how to find an app that would help.
☐	☐	I don't use apps at all.
☐	☐	I don't think I have a problem with stress, depression, anxiety or PTSD.
☐	☐	I am already in treatment for stress, depression, anxiety or PTSD and don't see the need for an app.
☐	☐	I am concerned about protecting my privacy with having my information in an app like this.
☐	☐	It would be embarrassing to have an app like this on my phone.
☐	☐	I tried an app like this before and did not like it because it was difficult to use.
☐	☐	I tried an app like this before and did not like it because it was not personalized enough.
☐	☐	I tried an app like this before and it did not help.

Below are some reasons why people may **decide to start** using mobile apps for stress, depression, anxiety or PTSD. Which reasons are true for you?

Yes	No	
☐	☐	I might use and app for these problems if I saw proof that it worked.

DOMAIN 6: Interest in Specific Mental Health App Features

Would you be interested in an app that allowed you to do any of the following?

Yes	No	
☐	☐	**I** Track your mood/stress/anxiety/PTSD symptoms.
☐	☐	Learn more about your mental health conditions.
☐	☐	Learn about how to change negative/self-critical thinking
☐	☐	Get involved in more activities.
☐	☐	Help you improve your social skills.
☐	☐	Help you learn to get better sleep.
☐	☐	Connect with a community of people with similar mental health problems.
☐	☐	Increase your physical activity or exercise.
☐	☐	Remind you to take your medications.
☐	☐	Speak with a health coach when your symptoms are bad.
